# Morphometric parameters predict body fat proportions in common hamsters

**DOI:** 10.1093/jmammal/gyab137

**Published:** 2021-11-25

**Authors:** Carina Siutz, Thomas Ruf, Stefanie Monecke, Eva Millesi

**Affiliations:** 1 Department of Behavioral and Cognitive Biology, Faculty of Life Sciences, University of Vienna, Djerassiplatz 1, 1030 Vienna, Austria; 2 Department of Interdisciplinary Life Sciences, Research Institute of Wildlife Ecology, University of Veterinary Medicine, Savoyenstraße 1, 1160 Vienna, Austria; 3 Institut des Neurosciences Cellulaires et Intégratives (INCI), Neurobiologie des Rythmes, CNRS UPR-3212, Université de Strasbourg, 8 allée du général Rouvillois, 67000 Strasbourg, France; 4 Institute of Medical Psychology, Medical Faculty, Ludwig-Maximilians-University Munich, Goethestrasse 31/ I, 80336 Munich, Germany

**Keywords:** body fat, common hamster, morphometrics, multiple regression, noninvasive, validation

## Abstract

Common hamsters (*Cricetus cricetus*) are hibernators that rely both on body fat reserves and food stores for the winter period. They face an ongoing population decline in most parts of their distribution and recently were classified as critically endangered. Knowledge on individual body fat proportions in this species is of particular interest for conservation, because it could contribute to better understand the high plasticity in overwintering strategies, overwinter mortality rates, individual variations in reproductive output, and give information on the animals’ health state. To calculate body fat proportions, we validated a method that can be applied in the field without the use of anesthesia. To develop this method, we first analyzed the body fat in carcasses of common hamsters using Soxhlet extractions and measured four morphometric parameters (body mass, head length, tibia length, foot length). The morphometric measurements were then integrated in a linear regression model to predict body fat proportions based on the measured values. The morphometric variables yielded an explained variance (adjusted *R*^2^) of 96.42% and body fat proportions were predicted with a mean absolute error of 1.27 ± 0.11% from measured values. We applied the model to predict body fat for available field data, which consistently produced reliable values. By measuring the four morphometric parameters and following the provided instructions, body fat proportions can be reliably and noninvasively estimated in captive or free-ranging common hamsters. Furthermore, the method could be applicable to other rodents after species-specific validation.

To escape energetic deficits, many animals living in seasonal climates hibernate during winter. Hibernation is a highly efficient energy-saving strategy, yet energy reserves are required to fuel fundamental metabolic demands. Most hibernators accumulate body fat reserves prior to winter, while others store food that can be used as external energy reserves (Humphries et al. 2003; Munro et al. 2008; Florant and Healy 2012; Sheriff et al. 2013). Common hamsters (*Cricetus cricetus*) are highly seasonal animals (Monecke et al. 2014b) and show a high plasticity in hibernation patterns (Wollnik and Schmidt 1995; Wassmer and Wollnik 1997; Monecke et al. 2011; Siutz et al. 2016, 2018a, 2018b). They are traditionally considered food-storing hibernators (Eibl-Eibesfeldt 1953; Niethammer 1982), but recent studies demonstrated sex differences in energy accumulation strategies in free-ranging hamsters (Siutz et al. 2012), indicating that in this species body fat reserves also play a prominent role for hibernation, particularly in males. Furthermore, prehibernation body mass appeared to affect hibernation performance under laboratory conditions (Siutz et al. 2017).

The common hamster, originally a typical steppe species, currently inhabits agricultural landscapes across Europe (Weinhold and Kayser 2006). It is invaluable for the ecological balance of agricultural and steppe ecosystems due to its role as prey of raptors and carnivores (Kayser et al. 2003; La Haye et al. 2020) and as predator of invertebrates and small rodents (Tissier et al. 2019), thereby regulating potential agricultural pest species. Farmland habitats, however, face a worldwide loss of biodiversity with declining numbers of many birds, invertebrates—including pollinators, and plant species (Wilson et al. 1999; Johst et al. 2006; Beketov et al. 2013; Stanton et al. 2018). This loss of biodiversity has been attributed to different aspects of agricultural intensification and its associated habitat loss, including increased mechanization, the use of pesticides, or the loss of landscape heterogeneity and, as a result, diverse food sources (Donald et al. 2001; Benton et al. 2003; Wilson et al. 2005; Butler et al. 2007; Millot et al. 2017; Stanton et al. 2018). Adverse effects of monocultures on population densities also were documented in mammal species, for example, European hares, *Lepus europaeus* (Smith et al. 2005). Accordingly, large monocultures and effective, quick, and early harvests represent serious threats for common hamsters, mainly because vegetation cover is virtually lacking, thereby increasing predation risk, and food availability often is unbalanced and temporally restricted (Kayser et al. 2003; Albert 2013; Tissier et al. 2016; 2017; La Haye et al. 2020). In the European Union, common hamsters are strictly protected since 1992 by the Bern Convention (Appendix II) and the Fauna-Flora-Habitat (Appendix IV) directives, because a strong population decline was observed in many Western European countries since the 1980’s (Weinhold 2008). Since about 20 years, various protection measures have been implemented, ranging from restructuring of large monocultures into smaller, differently cultivated fields, to the sowing of flower strips, and even to a complete abandonment of the harvest. In The Netherlands, France, Germany, and more recently Poland and Ukraine, wild populations are restocked by captive bred hamsters. Despite these protection efforts, however, populations could not be stabilized (Surov et al. 2016; Tissier et al. 2016). In 2020, the International Union for Conservation of Nature (IUCN) changed the status of the common hamster on its worldwide Red List by four categories from Least Concern to Critically Endangered (Banaszek et al. 2020).

The ongoing population decline in most parts of its distribution can be attributed, among others, to high overwinter mortality and a decline in seasonal reproductive output by 77% since the 1920’s (Kayser et al. 2003; Weinhold and Kayser 2006; Franceschini-Zink and Millesi 2008; Weinhold 2013; Surov et al. 2016). Information on whether fewer offspring are born or survive until independence is lacking. The reasons both for reduced overwinter survival rates and reproductive success, however, could partly be linked to insufficient energy reserves. Moreover, it was documented that the spring body mass of free-ranging hamsters in France decreased since the 1930’s by 21% (Tissier et al. 2016), but it is unknown if this is related to reduced body fat reserves or simply reduced size of the animals, an expected consequence of global warming. Developing and validating a method to noninvasively determine, or at least estimate, body fat proportions in common hamsters therefore could not only shed light on the variation in their hibernation performance and overwinter survival, but also could be crucial to the success of conservation activities. It would allow to identify critical periods in hamsters’ body condition, so that deficits in food or nutrients can be counteracted by, for example, nutritional improvements of the habitat or even food supplementation.

Several nondestructive methods to analyze body composition (e.g., lean body mass, body water, body fat) have been developed and applied in many different species. Two prominent techniques are total body electrical conductivity (TOBEC) and bioelectrical impedance analysis (BIA), which are based on the electrical characteristics of the body and provide direct estimates of body water and lean body mass, while body fat is determined indirectly (Speakman 2001). Both methods, however, yielded inconsistent results for predicting body fat: there were good estimates in some species (e.g., Hwang et al. 2005; Barthelmess et al. 2006; Pitt et al. 2006; Bergstrom and Sherry 2008), and quite inaccurate ones in others (e.g., Wirsing et al. 2002; Tidhar and Speakman 2007). Other methods, such as isotope dilution or dual-energy X-ray absorptiometry (DXA; Speakman 2001), also produced inconsistent results for body fat analyses similar to TOBEC and BIA (e.g., Nagy and Clair 2000; Korine et al. 2004; Tidhar and Speakman 2007; Johnson et al. 2017). In addition, these methods are relatively invasive because they involve either the injection of isotopes and blood sampling, or exposure to X-rays. Recent studies have used a quantitative magnetic resonance (QMR) technique, which mainly was applied to small animals (e.g., fish, mice, songbirds; Taicher et al. 2003; Tinsley et al. 2004; Guglielmo et al. 2011; Fowler et al. 2016; Kelsey and Bairlein 2019; Kelsey et al. 2020) but also a few larger ones such as laboratory rats (250–770 g; Johnson et al. 2009). In contrast to the other methods, this one does not require anesthesia and produces relatively precise body fat measurements, however, the analyzer optimally functions at room temperature and cannot be used in outdoor conditions (Taicher et al. 2003; Tinsley et al. 2004; Guglielmo et al. 2011; Kelsey and Bairlein 2019). Regardless of their accuracy, all methods mentioned above require special and in part expensive equipment.

Probably the most traditional and still commonly used approach to predict body fat is to apply morphological indices such as ratio or residual conditional indices (Hayes and Shonkwiler 2001) or scaled mass indices (Peig and Green 2009), which basically integrate measurements of body mass and size (e.g., body length, wing length, bill length). However, the usefulness of such indices was and still is a matter of debate (e.g., Schulte-Hostedde et al. 2005; Stevenson and Woods 2006; Labocha and Hayes 2012; Wilder et al. 2016) and will not be further discussed here. As an alternative to condition indices, computing multiple regression models which include morphological variables has been recommended and equations calculated from such models can be used to predict body fat (Hayes and Shonkwiler 2001; Labocha and Hayes 2012; Labocha et al. 2014).

Here we present a method to noninvasively predict body fat proportions in common hamsters, based on a multiple regression model. We aimed at developing a method that can be applied easily in the field without the use of special equipment and, most importantly, without anesthesia that would require (apart from authorized persons) permitting obtained with difficulty in this endangered species. We used measurements of four morphological parameters: body mass, head length, tibia length, and foot length, all of which can be obtained reliably in the laboratory as well as in the field without the use of anesthesia. We measured body fat mass using chemical extractions (Soxhlet) as well as the morphological parameters in carcasses of common hamsters. We previously attempted to develop and apply this method (Siutz et al. 2012); that study, despite yielding good results, suffered from an insufficient sample size (*n* = 11 carcasses) to reliably predict body fat in a broader range of individuals. In the present study, we substantially increased the sample size. The predictive power (explained variance, *R*^2^) of the morphological parameters for body fat proportions was determined by calculating a linear multiple regression model. In addition, we provide the data used to compute the model that enables the prediction of body fat proportions in any living common hamster if the above listed morphological parameters are available.

## Materials and Methods

### Ethical statement.

The hamsters originated from the Chronobiotron laboratory breeding stock, Strasbourg, France. Supernumerary juveniles and disused breeding individuals were euthanized. Animals of less than 160 g body mass were anesthetized by isoflurane and euthanized by cervical dislocation. Heavier animals were anesthetized and euthanized with CO_2_ (Authorization B-67-482-25). The carcasses of these hamsters were obtained for this study.

### Study animals.

All hamsters were untreated and intact prior to anesthesia. After euthanization, carcasses were immediately stored individually in airtight freezing bags at −20°C. Carcasses were transported in cool boxes equipped with ice packs and arrived at our laboratory without thawing. Body fat extractions were carried out about six months after initial storage. We used 74 carcasses for analyses, including juveniles and adults of both sexes. Age ranged from 17 days to 39 days in juveniles (*n* = 42) with body mass ranging from 55 g to 175 g. Adult individuals (*n* = 32) were between 12.7 and 14.4 months old and body mass ranged from 209 to 559 g. We primarily chose these individuals to approximately cover the full range of body mass we measured in free-ranging hamsters at our field study sites (i.e., from about 50 g in juveniles to about 600 g in adults). Due to the limited availability of carcasses, however, this entailed a variable sex ratio within age classes.

### Morphological measurements.

As a proxy for body size, we measured the following morphological parameters: (1) head length, measured as length from tip of the nose to the posterior edge of the occipital bone with the head being in a straight position; (2) tibia length, measured as length from top of the knee to bottom of the heel bone in the left hind leg with the lower leg being rectangular to the thigh; (3) foot length, measured as length from the posterior edge of the heel bone to the top of the middle toe (without claw) in the left hind foot with the foot being in a straight position. In contrast to, for example, body length, these parameters can be measured in living and free-ranging hamsters without the use of anesthesia by handling the animal in a cone-shaped cotton sack laterally equipped with a Velcro fastener (e.g., Franceschini et al. 2007), which enables investigation of the abdominal region while the head is fixed in the front part of the sack. Hamsters usually keep very calm during handling in these sacks, which enables precise measurements of the morphological parameters. In addition, the sack has a small opening in the front uncovering the snout and a slit at the approximate position of the occipital bone to accurately measure the head length (see figures in Supplementary Data SD1).

To obtain accurate measurements, each parameter was measured at least three times per individual using an electronic sliding caliper (± 0.1 mm). For analyses, mean values were calculated from three measurements if the deviation from the mean of a single measurement was < 0.5 mm. Otherwise, the measurement was repeated another two times to obtain three measurements with a deviation < 0.5 mm from the mean. Finally, body mass was recorded using an electronic scale (± 1 g). All measurements were taken in thawed carcasses immediately before body fat extraction and were undertaken by the same person (CS).

### Body fat extraction.*—*

We used whole carcasses (including fur and intestines) for analyses because our aim was to validate a method applicable to free-ranging animals in which only total body mass can be recorded and both stomach/gut contents and molt are uncontrollable. The use of all components of an animal also was recommended previously (Reynolds and Kunz 2001). Carcasses were minced using a commercial meat grinder and thoroughly homogenized. After drying for 16 h at 100 °C, body fat was determined by lipid extraction using a Soxhlet apparatus with petroleum ether as solvent. Total body fat was weighed ± 0.001 g and its percentage of total wet mass was calculated.

### Statistics.

Statistical analyses were undertaken in R (R Development Core Team 2018). The linear model included measured body fat proportions as response variable and measured body mass, head, tibia, and foot length, as predictor variables as well as their interactions. Predicted values for body fat proportions were obtained by using the “predict” command (package “stats”), which is a generic function for predictions from model results.

To predict body fat proportions as accurately as possible, we aimed at computing a model that, on one hand, yielded a high explained variance (adjusted *R*^2^), a minimal absolute error (|measured-predicted|), and a high correlation coefficient (*r*) of predicted and measured fat values, but on the other hand, also produced reliable predicted fat values (i.e., body fat proportion of 0 – 45%, where the maximum value was set based on a study in a fat-storing hibernator, the arctic ground squirrel, showing mean fat proportions of up to 41.5 ± 2%; Buck and Barnes 1999). The initial model included all possible interactions between the predictor variables, which yielded an adjusted *R*^2^ = 97.16%, absolute error of 1.08 ± 0.11%, and *r* = 0.99, but generated negative and, hence, unreliable predicted fat values, which could have indicated overfitting. Therefore, we stepwise eliminated interactions beginning with the 4-way interaction followed by the 3-way interactions. For each model, we checked the statistical power (*R*^2^, absolute error, *r*) and the predicted fat values (reliability). The final (predictive) model included all possible 2-way interactions between the predictor variables and had an adjusted *R*^2^ similar to the initial one, but produced reliable predicted fat values (see Results section). Model residuals were normally distributed as revealed by a Shapiro-Wilk test and visual inspection of quantile–quantile plot. Descriptive values in the Results section are presented as means ± *SE*.

Data of measured morphological parameters and body fat proportions as well as all statistical commands including detailed instructions necessary for predicting body fat proportions in any living common hamster are provided as Supplementary Data (Supplementary Data SD2: measured morphological parameters and body fat proportions; Supplementary Data SD3: statistical commands and instructions).

To cross-validate the method, we applied three approaches: first, we created a training data set that contained randomly chosen 70% (*n* = 52) of the carcasses. This data set was used to generate a regression model including the same predictor variables as the predictive model. The training model then was applied to the testing data set, which comprised the remaining 30% (*n* = 22) of the carcasses. This approach enabled us to implement the method on an independent sample and compare predicted body fat proportions to measured ones. Second, we applied the predictive model to a completely independent sample of individuals (*n* = 11), which were found dead in the field (without any signs of decay or obvious injuries/diseases) and for which both measured morphological parameters and body fat proportions were available. The procedure of body fat extraction was the same as described here, but the carcasses were stored at -20 °C for 1–5 years before analysis. Third, we implemented the predictive model to morphometric parameters measured in free-ranging hamsters at our field sites in southern Vienna, Austria, throughout the active season (March—October) in different years. Data on 350 individuals across all sex and age groups (175 juveniles and 175 adults) were chosen randomly but included the smallest/lightest and largest/heaviest individuals of our data set. Although measured body fat proportions were not available in this data set, we intended with this approach to ensure that the predictive model produced sensible values for body fat proportions (i.e., > 0% and < 45%; see above) and that the method could be applied to alive common hamsters.

## Results

### Predictive model.

Body fat proportions measured in carcasses ranged from 0.94% to 34.34% (10.94 ± 0.99%) of total wet mass (see Supplementary Data SD2). The most robust linear regression model (maximizing *R*^2^ while restricting predictions to realistic positive values) revealed significant main effects of body mass, head length, and tibia length, as well as significant interaction effects on body fat proportions of body mass:tibia length, head length:foot length, and tibia length:foot length (Table 1), with body mass showing the strongest effect, followed by tibia length and the interaction between body mass and tibia length.

**Table 1.** ANOVA (Type III) table for effects of body mass, head, tibia, and food length as well as their 2-way interactions on measured body fat proportions (response variable) in carcasses of common hamsters.

Predictor variable	*F*-value	*P*-value
Body mass (g)	40.12	<0.001
Head length (mm)	7.79	0.007
Tibia length (mm)	13.23	0.001
Foot length (mm)	0.93	0.34
Body mass: head length	1.44	0.24
Body mass: tibia length	13.41	0.001
Body mass: foot length	0.29	0.59
Head length: tibia length	0.88	0.35
Head length: foot length	11.8	0.001
Tibia length: foot length	4.07	0.048

The formula to calculate body fat proportions is (all lengths in mm):

*body fat proportion (%) = 0.323764 × BM—3.708651 × HL + 4.955313 × TL—1.256918 × FL + 0.003047 × BM: HL—0.006242 × BM: TL—0.001737 × BM: FL—0.027821 × HL: TL + 0.155368 × HL: FL—0.140722 × TL: FL*

where BM is body mass, HL is head length, TL is tibia length and FL is foot length.

**Fig. 1.**
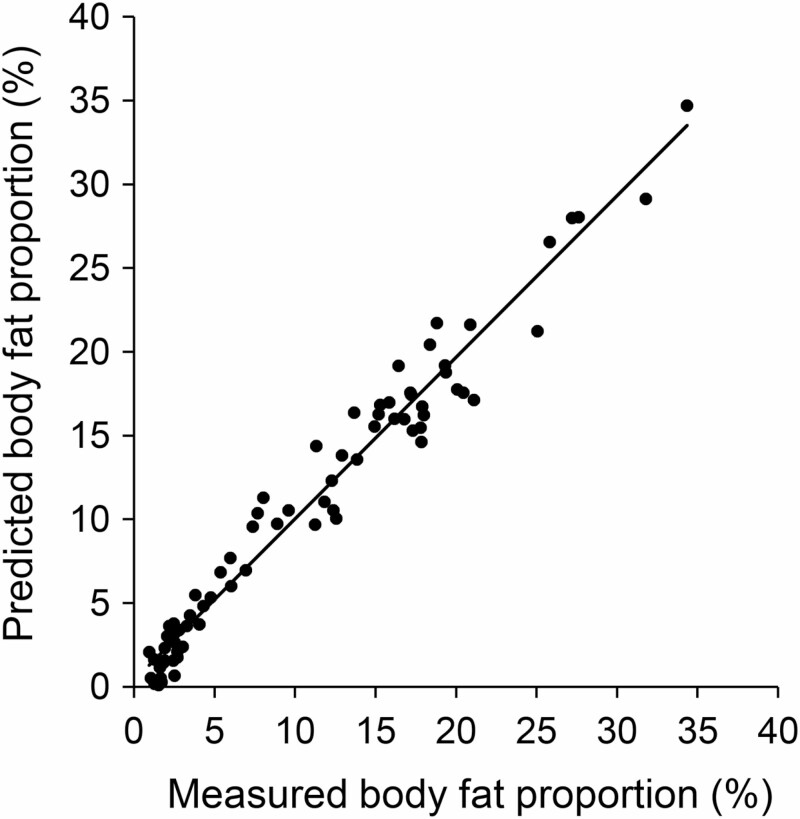
**—**Predictability of body fat proportions. Relationship between measured and predicted values of body fat proportions (*n* = 74, *r* = 0.98, *P* < 0.001) based on a linear regression model (adjusted *R*^2^ = 96.42%, mean absolute error = 1.27 ± 0.11%) integrating the morphometric parameters body mass, head, tibia, and food length as well as their 2-way interactions measured in carcasses of common hamsters.

This model yielded an adjusted *R*^2^ of 96.42% (*F*_10,64_ = 472.1, *P* < 0.001) and predicted body fat proportions with a mean absolute error of 1.27 ± 0.11% (min: 0.001%, max: 3.99%) of measured values, although the absolute error was below the mean in 61% of the cases. Moreover, the model almost equally underestimated body fat proportions in 46% (actual error: -1.38 ± 0.19, min: -0.02%, max: -3.99%) and overestimated it in 54% of cases (actual error: 1.17 ± 0.14, min: 0.001%, max: 3.27%). The predicted body fat proportions, ranging from 0.1% to 34.7% (10.94 ± 0.97%), correlated highly with the measured ones (*r* = 0.98, *P* < 0.001; Fig. 1).

### Cross-validation.

**Fig. 2.**
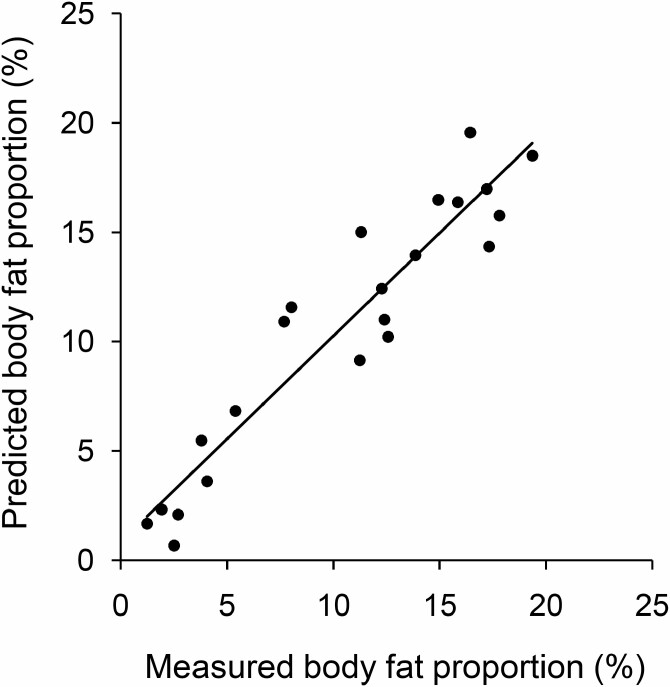
**—** Relationship between measured and predicted values of body fat proportions (*n* = 22, *r* = 0.94, *P* < 0.001) of the testing data set (30% of carcasses) revealed by applying the training model (70% of the carcasses), which predicted body fat proportions with a mean absolute error of 1.58 ± 0.25% of measured values.

The regression model generated by the training data set (data of 70% of the carcasses; *n* = 52) yielded an adjusted *R*^2^ of 97.45% (*F*_10,42_ = 396.5, *P* < 0.001). Implementing this model on the testing data set (remaining 30% of the carcasses; *n* = 22) predicted body fat proportions with a mean absolute error of 1.58 ± 0.25% of measured values. The actual errors averaged 0.25 ± 0.42% and ranged from -2.95% to 3.72% (Fig. 2). Measured body fat proportions in carcasses found in the field (*n* = 11) ranged from 0.86% to 18.36%. Applying the predictive model to these individuals resulted in actual errors between -2.68% and 3.86% from measured values. The mean absolute error was 2.25 ± 0.35%.

**Fig. 3.**
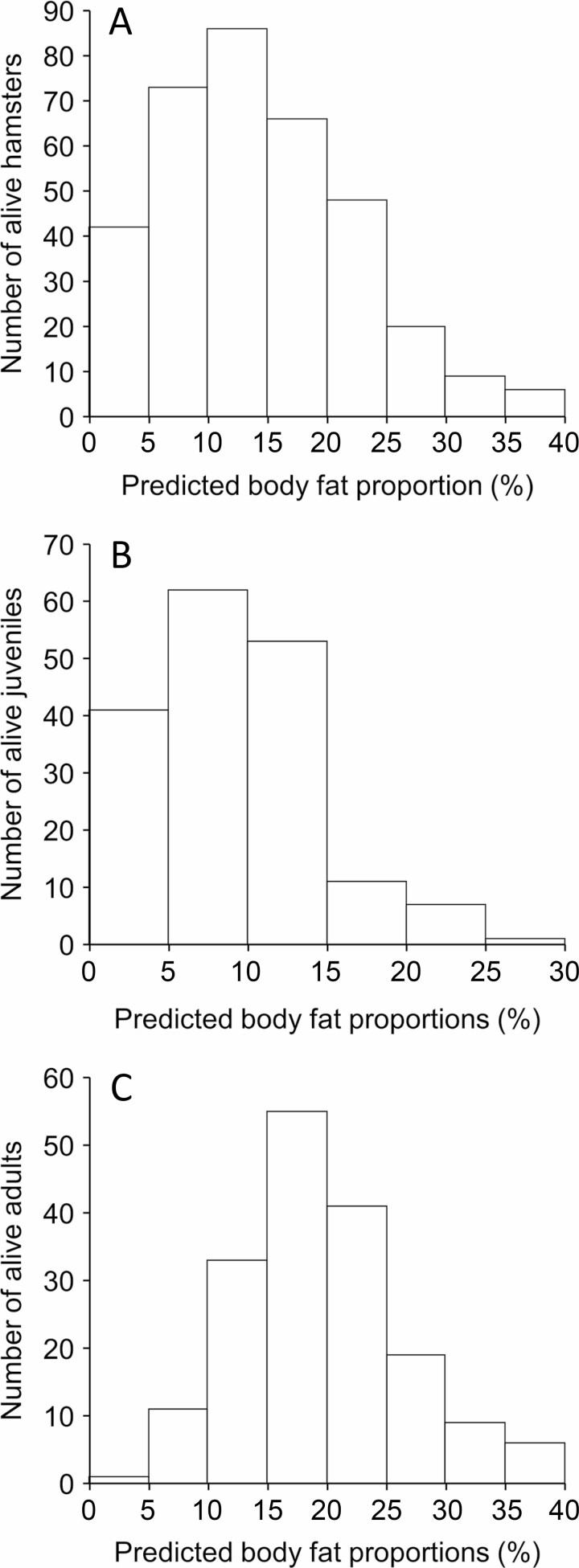
**—** Frequency distribution of predicted body fat proportions in free-ranging common hamsters (*n* = 350) across all age and sex groups (A), across juvenile (*n* = 175) individuals (B), and across adult (*n* = 175) individuals (C) sampled in Vienna, Austria, during the active season (March to October) in different years.

Using the predictive model on morphological measurements of 350 randomly chosen free-ranging common hamsters revealed predicted body fat proportions ranging from 0.06% to 36.76%. Most individuals (64%) showed predicted body fat proportions between 5% and 20% (Fig. 3A). Body fat proportions of ≤ 5% only were found in juveniles, with the exception of two adult females. Similarly, proportions of ≥ 25% only were found in adult hamsters as well as in one juvenile. In general, the majority (89%) of juveniles had body fat proportions of < 15% (Fig. 3B), including 8 individuals (5%) with proportions of < 1%, while most adult hamsters (74%) showed body fat proportions ranging from 10% to 25% with 6 individuals (3%) exceeding proportions of 35% (Fig. 3C).

## Discussion

Our principal objective was to develop and validate a convenient method to estimate body fat in common hamsters that can easily be applied both to captive and free-ranging individuals without the use of anesthesia. Using multiple regression models based on morphological measurements is a commonly employed method to predict body fat, although the explanatory power of the morphological parameters showed considerable variation among various studies and is usually quite low (Labocha and Hayes 2012; Labocha et al. 2014; Fowler et al. 2018; McGuire et al. 2018; Zaniewicz et al. 2018). Our predictive regression model included not only single morphological parameters but also their interactions and, accordingly, yielded a remarkably high explained variance, validating the morphological parameters for predicting body fat. Such, high explained variances achieved by morphological measurements have been found in some bird species, although these models also included visual fat scores (Bergstrom and Sherry 2008; McWilliams and Whitman 2013) or abdominal fat deposits (Fowler et al. 2018). Crucially, our regression model predicted body fat proportions with a high accuracy because the mean absolute error from measured values was approximately 1% and even below the mean in about 60% of the cases. Moreover, the cross-validation approach of generating a training model and applying it to a testing data set reflected the results obtained by the predictive model. Likewise, the body fat proportions calculated by the predictive model on carcasses found in the field differed from the measured body fat proportions in these individuals only by ca. 2%. This further supports the high accuracy of body fat proportions derived by using the predictive model. Finally, our method has the additional advantage that body fat proportions can be computed easily from new morphological measurements using the provided Supplementary Data SD2 and the “predict” function in R, although manually calculating fat proportions based on the formula above also is accurate.

Some studies included age classes and/or sex as predictor variables in the regression models or calculated separate models for males and females (e.g., Tidhar and Speakman 2007; Fowler et al. 2018). Our model is not age- or sex-specific despite the differences in morphological measurements between juveniles and adults as well as the sexual dimorphism in common hamsters, with males being larger and heavier than females (Niethammer 1982; Grulich 1987; Petrová et al. 2018; Kryštufek et al. 2020). Although the predictability of body fat could have been more accurate when generating separate models for juveniles and adults, combining age classes has the advantage of improving the applicability of the model to field studies. Free-ranging juveniles, subadults (before first hibernation), yearlings (after first hibernation), and adults (after second hibernation) cannot always be clearly distinguished based on body mass or size because growth rates of juveniles can differ by much, e.g., due to the season of birth (Kirn 2004) and whether they have reached puberty or not (Monecke et al. 2014a). Body mass at the end of the season therefore can be similar between subadults and yearlings as well as yearlings and adults (Vohralík 1975; Lebl and Millesi 2008; Müskens et al. 2011; Pluch et al. 2013; Petrová et al. 2018; Kryštufek et al. 2020). To deal with sexual dimorphism, we used male and female carcasses with a high variation in body mass and size, resulting in a broad overlap in all morphometric parameters between the sexes. For example, although the heaviest and largest individual used to generate the model was an adult male, body mass of 50% of adult males was within the body mass range of adult females and head length of 75% of adult males was within the head length range of adult females.

One limitation of regression models is that they often are not applicable to different populations or across seasons because individuals can differ in morphological traits depending on their geographic origin, and the accumulation of body fat reserves could be season-specific (Labocha and Hayes 2012; Fowler et al. 2018). However, we are confident that our model can be used to predict body fat proportions in any living hamster for three reasons: first, we used carcasses with a relatively wide range of morphological measurements, both within sex and age classes, to generate the regression model, which, similar to Bergstrom and Sherry (2008), substantially increased the explained variance in the data set. Second, morphological measurements in carcasses were similar to individuals at our field site, but the carcasses were obtained from a breeding colony and represented not only a different population but even a different phylogeographic lineage (Vienna wild populations belong to the Pannonian lineage, the Strasbourg breeding colony to the North lineage; Neumann et al. 2005; Banaszek et al. 2010). Third, although common hamsters are hibernators, they show only moderate fattening prior to winter, as indicated by body mass changes (Lebl and Millesi 2008; Hufnagl et al. 2011), resulting in lower seasonal variation in body fat reserves compared to other hibernating species. Caution is strongly warranted, however, if body fat proportions are to be predicted in individuals whose morphological measurements are not within the range of those used to calculate the predictive model (see Supplementary Data SD2). In such cases, predicted body fat proportions are likely to be under- or overestimated.

To test the applicability of the method in living hamsters, we implemented the model to available data from 350 specimens of free-ranging common hamster. This proved successful given that sensible values (i.e., not negative or greater than 45%) were obtained. The significance of these results cannot be fully interpreted because the data only represent single sampling points and a comprehensive picture of all available data of an individual would be necessary. We can, however, provide some descriptive information. Juveniles sampled in this data set showed body fat proportions mainly ranging from 5% to 15%, although body fat proportions were below 5% in about one quarter of individuals. These were primarily juveniles with a body mass of less than 100 g sampled shortly after natal emergence and could reflect the fat proportions at this age. The lowest value of 0.06% body fat was found in a juvenile which had a body mass 4 g below the lower limit of the body mass range in individuals used to calculate the predictive model; body fat therefore might have been underestimated in this case. Juveniles with more than 20% body fat were sampled later in the season, partly shortly before winter, and probably had sufficient time to accumulate fat deposits. Two adult females showed body fat proportions of 4.4% and 5%, respectively. The first female was sampled after her second lactation period and the second female immediately after winter and, hence, could represent possible fat proportions during such periods. In general, adult hamsters with less than 15% body fat all were sampled either after winter, during the mating period (males), or during or after lactation (females). In contrast, body fat proportions greater than 30% all were found in males as well as two females sampled shortly before winter and both had relatively high body mass for females (445 g and 524 g, respectively). Taken together, these data demonstrate the applicability of our method in field studies, for instance, to identify critical periods during the active season.

For applying our model to predict body fat proportions in common hamsters, we recommend measuring the morphological parameters as precisely as possible. This includes at least three consecutive measurements of each parameter, which should be within a deviation of 0.5 mm and ideally taken by the same person. To avoid anesthesia, hamsters can be handled in cotton sacks, in which they usually keep very calm, thereby enabling exact measurements; this might require some practice, particularly to ensure the correct position of the head. In agitated individuals, however, we recommend measuring a morphological parameter at least five times. In addition, the measurements also can be taken on anesthetized animals. Finally, it certainly must be assured that a female is not in a late gestational stage because this would strongly confound body mass measurements.

In conclusion, our results support the use of multiple regression models based on morphological measurements as a practical, appropriate, and in particular, an accurate method to estimate body fat proportions. Including interaction terms of the predictor variables in the model resulted in precise predictions of body fat proportions; using the “predict” function in R makes our approach even more convenient. Applying our method not only allows one to investigate effects of body fat reserves on hibernation performance but also the relationship of body fat with overwinter survival and reproductive success. Internal energy reserves of females as well as the number of pups they successfully raise are greatly affected by the nutritional composition of their diets, and malnutrition can lead to maternal infanticide (Tissier et al. 2017). Moreover, it has been shown that hamster populations can survive in hamster-friendly managed fields only if harvest is delayed until early August (which is far later than in regular managed fields) and the mean litter size is seven (La Haye et al. 2014). Understanding the nutritional needs of common hamsters for optimal reproductive success therefore might be the missing link to counteract the population declines in this species. The method presented here offers the possibility to monitor body fat reserves of free-ranging common hamsters living under different nutritional conditions. In combination with diet reconstructions by stable isotope analysis of hair samples (Roswag et al. 2018) it could help to nutritionally improve habitats for common hamsters and thereby also for other species. Furthermore, after species-specific validation of morphological characters, the principles of our method are applicable across many other species. This method therefore represents an important tool, particularly for applications in wildlife conservation programs.

## Supplementary Material

Supplementary data are available at *Journal of Mammalogy* online.

Supplementary Data SD1.—Pictures of measuring the morphological parameters and animal handling in a cotton sack. (A-B) Head length was measured from tip of the nose to the posterior edge of the occipital bone; (C-D) Tibia length was measured from top of the knee to bottom of the heel bone with the lower leg being rectangular to the thigh; (E-F) Foot length was measured from the posterior edge of the heel bone to the top of the middle toe (without claw); (G-H) For animal handling without anesthesia, the hamster is released into a cotton sack; the sack is cone-shaped, hence, fixing the head in the front part of the sack; a small opening in the front uncovering the snout and a slit at the position of the occipital bone allow to accurately measure the head length (see also A); opening the Velcro fastener enables measuring tibia and foot length (see also D and F).

Supplementary Data SD2.—Data file used to compute the model including morphological parameters and body fat proportions measured in carcasses of common hamsters. If using the statistical software R to predict body fat proportions in any living common hamster based on our model, this data file needs to be read by the software.

Supplementary Data SD3.—Instructions for predicting body fat proportions using the statistical software R. This file includes all statistical commands necessary for predicting body fat proportions in any living common hamster.

## Supplementary Material

gyab137_suppl_Supplementary_Data_SD1

gyab137_suppl_Supplementary_Data_SD2

gyab137_suppl_Supplementary_Data_SD3
