# Supplementary material for: Morphometric parameters predict body fat proportions in common hamsters
Source: J Mammal. 2021 Nov 25;103(2):471–80. doi: 10.1093/jmammal/gyab137 (PMC8996034; doi:10.1093/jmammal/gyab137)
Supplement: gyab137_suppl_Supplementary_Data_SD1 [file gyab137_suppl_supplementary_data_sd1.pdf]

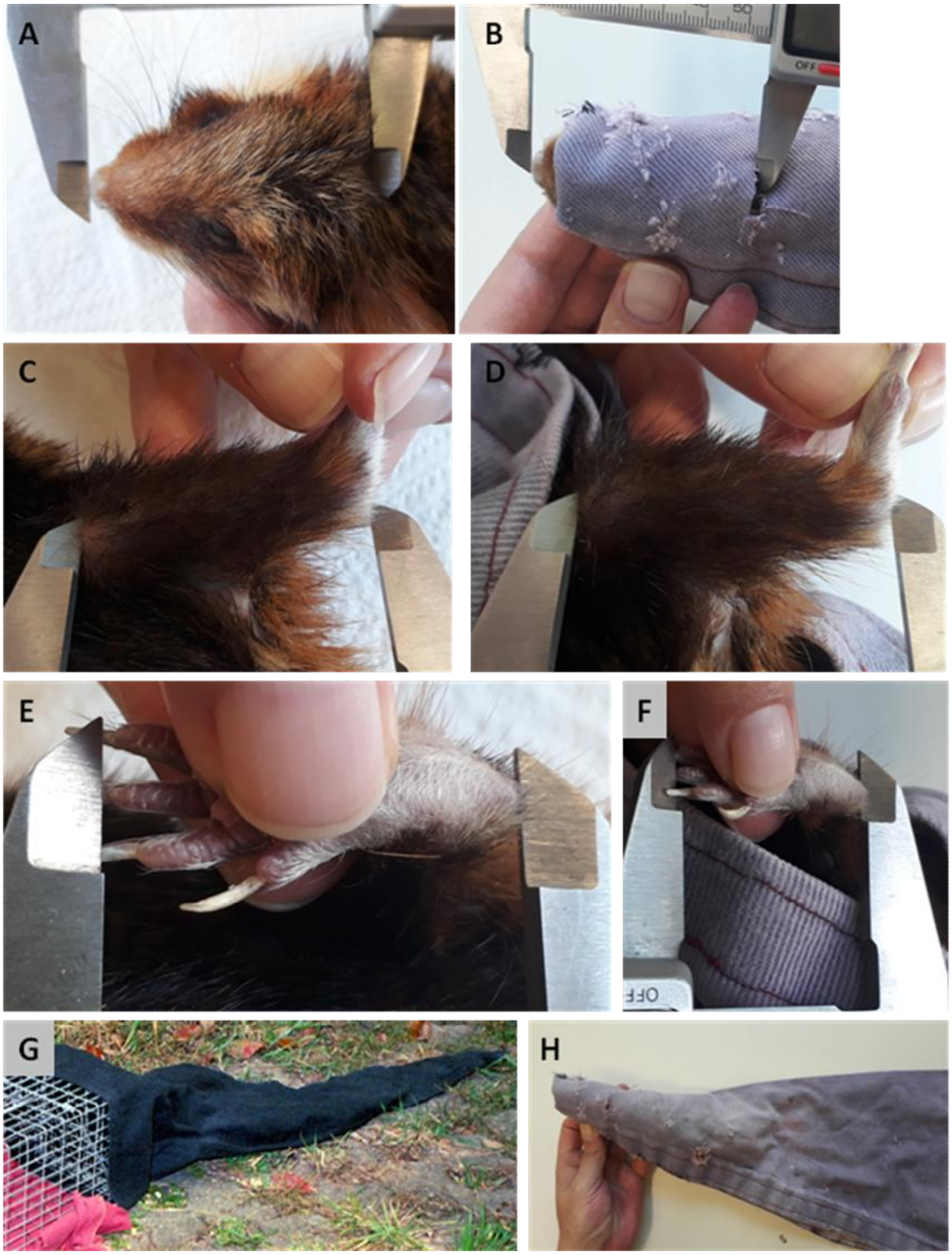

Supplementary Data SD1.—Morphometric measurements and animal handling in a cotton sack. (A-B) Head length was measured from tip of the nose to the posterior edge of the occipital bone; (C-D) Tibia length was measured from top of the knee to bottom of the heel bone with the lower leg being rectangular to the thigh; (E-F) Foot length was measured from the posterior edge of the heel bone to the top of the middle toe (without claw); (G-H) For animal handling without anesthesia, the hamster is released into a cotton sack; the sack is cone-shaped, hence, fixing the head in the front part of the sack; a small opening in the front uncovering the snout and a slit at the position of the occipital bone allow to accurately measure the head length (see also B); opening the Velcro fastener enables measuring tibia and foot length (see also D and F).
